# Functional electrical stimulation following anterior cruciate ligament reconstruction: a randomized controlled pilot study

**DOI:** 10.1186/s12984-019-0566-0

**Published:** 2019-07-12

**Authors:** Uria Moran, Uri Gottlieb, Arnon Gam, Shmuel Springer

**Affiliations:** 1 Israel Defense Forces Medical Corps, Ariel, Israel; 2 Department of Physical Therapy, Faculty of Health Sciences, Ariel University, 40700 Ariel, Israel

**Keywords:** Anterior cruciate ligament, Reconstruction, Functional electrical stimulation, Neuro-muscular electrical stimulation

## Abstract

**Background:**

Inadequate quadriceps strength following anterior cruciate ligament reconstruction (ACLR) often results in alterations in gait pattern that are usually reported during loading response. Neuro-muscular electrical stimulation (NMES) is frequently used to overcome this quadriceps weakness. Despite the beneficial effects of NMES, persistent deficits in strength and gait are reported. The aim of this study was to investigate the feasibility of applying quadriceps functional electrical stimulation (FES) during walking in addition to standard rehabilitation, in the initial stage of ACLR rehabilitation.

**Methods:**

Subjects were randomized to quadriceps FES synchronized with walking group (*n* = 10) or quadriceps NMES (duty cycle of 10 s on/10 s off) group (*n* = 13). Both interventions were performed for 10 min three days a week, in addition to a standard rehabilitation program. Assessments were performed up to 2 weeks before the ACLR (pre-ACLR), and 4 weeks postoperatively. Outcomes measured were gait speed, single limb stance gait symmetry, quadriceps isometric peak strength ratio (peak strength at 4 weeks/peak strength pre-ACLR) and peak strength inter-limb symmetry. Gait outcomes were also assessed 1-week post-surgery.

**Results:**

Subjects in both groups regained pre-ACLR gait speed and symmetry after 4 weeks of rehabilitation, with no difference between groups. However, although pre-ACLR quadriceps peak strength was similar between groups (FES - 205 Nm, NMES − 225 Nm, *p* = 0.605), subjects in the FES group regained 82% of their pre-quadriceps strength compared to 47% in the NMES group (*p* = 0.02). In addition, after 4 weeks, the FES group had significantly better inter-limb strength symmetry 0.63 ± 0.15 vs. 0.39 ± 0.18 in the NMES group (*p* = 0.01).

**Conclusions:**

Quadriceps FES combined with traditional rehabilitation is a feasible, early intervention treatment option, post-ACLR. Furthermore, at 4 weeks post-surgery, FES was more effective in recovering quadriceps muscle strength than was NMES. While spatiotemporal gait parameters did not differ between groups, kinetic and kinematic studies may be useful to further understand the effects of quadriceps FES post-ACLR. The promising results of this preliminary investigation suggest that such studies are warranted.

**Trial registration:**

ISRCTN 02817399. First posted June 29, 2016.

## Introduction

People who undergo anterior cruciate ligament reconstruction (ACLR) often experience quadriceps muscle weakness [1, 2]. This muscle weakness is frequently due to arthrogenic muscle inhibition (AMI), a term that describes the inability to completely contract a muscle despite no structural damage to the muscle or innervating nerve [3]. Long-term deficits in strength are reported post-surgery, even after the formal rehabilitation period ends; leading to inability to return to preinjury level of sports and physical activity and potential for reinjury [1, 2].

Inadequate quadriceps strength following ACLR often results in persistent alteration in gait pattern, usually during loading response. During this phase of the gait cycle, the limb accepts full support of the body towards the single limb stance. To absorb impact, the knee moves into flexion, which is controlled by eccentric contraction of the quadriceps. ACLR subjects were found to have deficits in knee flexion range of movement and reduction in knee extensor moment [4–6], resulting in an asymmetrical gait pattern. Furthermore, it was shown that gait asymmetry does not appear to normalize over time, despite return to physical activity [5, 7], and that the gait pattern of subjects with weak quadriceps post-ACLR resembles that of acute ACL deficiency, despite surgical restoration of knee stability [4, 8].

Gait speed is another important indicator of recovery after ACLR. Walking speed is a good measure of energy, motor control, endurance and muscle function; Moreover, it has been deemed the “sixth vital sign,” as it is a predictor of future and present health status, and potential response to rehabilitation [9]. Due to altered gait pattern, reduced gait speed is commonly reported in individuals after ACLR [10]. Furthermore, slower walking speed and reduced muscle control during the loading response of the gait cycle has been correlated with greater collagen breakdown post-ACLR and higher risk to develop knee osteoarthritis [11].

Neuromuscular electrical stimulation (NMES) applied to the quadriceps is frequently used to overcome quadriceps weakness following ACLR [12–15]. NMES may facilitate recruitment of the muscle that is inhibited by AMI [13]. Exercise combined with NMES was shown to be more effective in improving quadriceps strength than was exercise alone following ACLR [13]. A recent systematic review concluded that NMES in addition to standard physical therapy appears to significantly improve quadriceps strength and physical function in the early post-operative period compared to standard physical therapy alone [12]. Nonetheless, despite the beneficial effects of NMES, deficits in strength are still reported [13]. For example, in study by Lepley et al. [14] subjects received NMES two times per week for 6-weeks following ACLR; Yet, after 12- weeks, there was still 33% deficit in quadriceps strength compared to pre-intervention.

Functional electrical stimulation (FES) is an alternative method of applying electrical stimulation for muscle strengthening and motor recovery. When stimulation is employed during the performance of specific tasks or during daily functions, the term FES rather than NMES is a more accurate description of the application. The unique characteristics of FES, which provide stimulation in a coordinated, rhythmic pattern with the targeted movement, contribute to recovery of muscle control [16]. Furthermore, FES incorporates neuromuscular training with motor learning principles which optimize quadriceps strengthening following ACLR [17]. Accordingly, application of FES to the quadriceps muscle during gait may enhance its function in the early post-operative phase. This could lead to more symmetrical loading of the legs during walking. While quadriceps FES has been used to improve gait performance in subjects with hemiparesis [18], no previous study has tested the feasibility of applying FES during gait as a method of enhancing quadriceps muscle strength and motor recovery following ACLR or other musculoskeletal conditions.

Therefore, the objectives of this study were to investigate the feasibility of adding quadriceps FES during walking to the standard ACLR rehabilitation program and to test the effectiveness of this method on gait and on quadricep muscle strengthening during the initial stage of ACLR rehabilitation, compared to NMES combined with standard rehabilitation.

## Methods

### Participants

All participants were candidates for minimally invasive reconstruction of the ACL. Participants were screened, recruited, and underwent rehabilitation at the Military Rehabilitation Center, Zrifin, Israel. Recruitment and data collection were occurred from May 2016 to April 2017. Inclusion criteria were: age 18 to 40 years; ACL reconstruction using a graft from the patellar, semitendinosus or gracilis tendon; and ability to comply with the rehabilitation protocol (i.e., attend treatment 3 days a week at a clinical rehabilitation site). Exclusion criteria were previous injury or surgery of the injured knee; major injury to the lower limb in the 2 years prior to the ACLR (e.g., fracture, rupture of Achilles tendon); or history of chronic ankle instability.

Initially, 97 subjects were approached to participate in the study. Among this group, 57 (58.8%) did not meet the inclusion criteria. Forty patients were eligible for the study and were randomized to FES or the NMES group according to a computer-generated schedule, matched for age. The sample size of 40 participants was based on previous pilot studies describing ACLR rehabilitation [19–21], as well as on the expected number of potentially eligible patients undergoing ACLR who would be accessible for the duration of this pilot study. Recruitment was stopped when the targeted sample size was met.

**Fig. 1:**
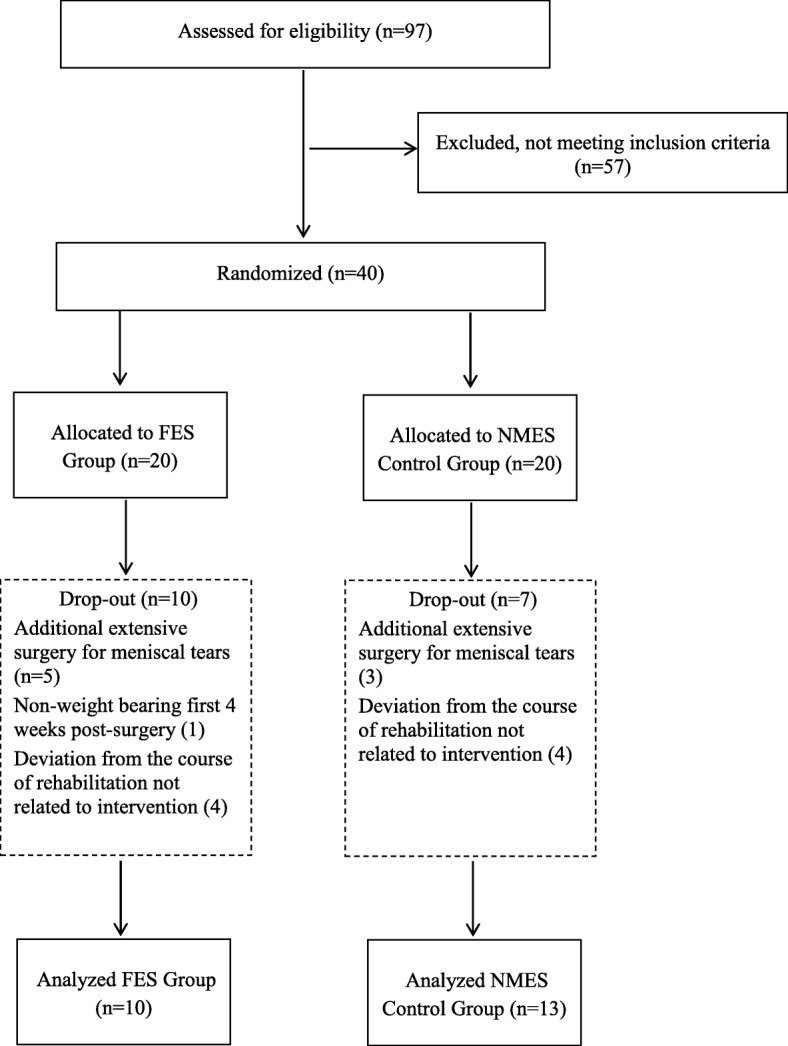
Flowchart of the patients recruited for the study

Seventeen patients later dropped out or were excluded from the study for reasons that included, additional extensive surgical treatment of meniscal tears on the involved knee, instruction for non-weight bearing for 4 weeks post-surgery, or not complying with the course of rehabilitation for reasons unrelated to the intervention. Consequently, 23 patients completed all protocol and testing sessions and were included in the data analysis (Fig. 1).

The study was approved by the Israel Defense Force Medical Corps Ethics Review Board (approval number IDF-1602-2015) and was registered at ClinicalTrials.gov (Identifier: NCT02817399). All participants provided written informed consent to participating in the study.

### Standardized postoperative rehabilitation protocol

Patients from both groups participated in a standard, postoperative rehabilitation protocol, supervised by a physical therapist, as suggested by Adams et al. [22].

The main postoperative milestones were Week 1: active/passive knee range of motion (ROM) 0° to 90°; Week 2: knee flexion greater than 110°, walking without crutches, ability to use a cycle/stair climber without difficulty, reciprocal stair climbing, straight leg raising without extension lag; Week 3–4: knee flexion ROM to within 10° of uninvolved side, quadriceps strength at least 60% of uninvolved side.

### Electrical stimulation protocols

**Fig. 2:**
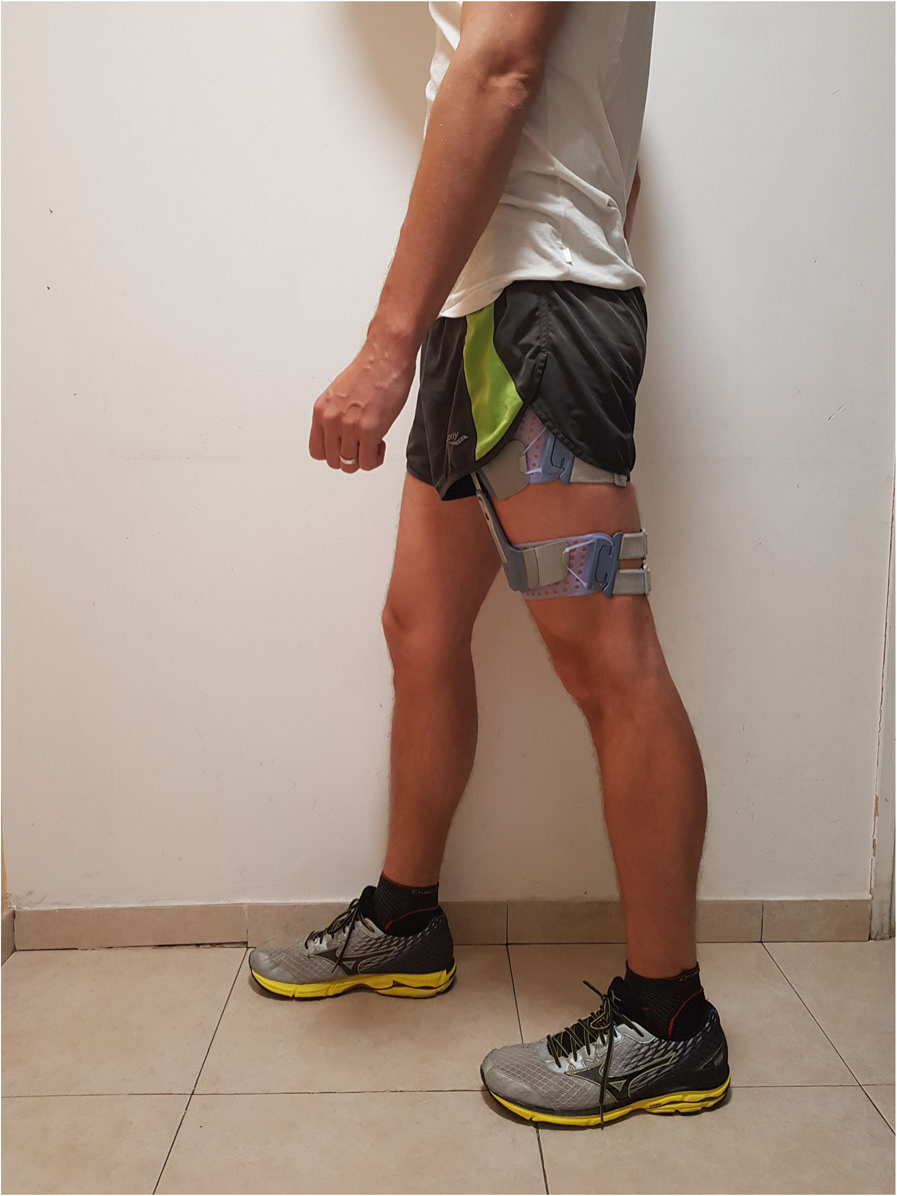
The electrical stimulation system used in the study

The electrical stimulation system used in this study (NESS L300Plus, Bioness, Valencia, CA) enables application of NMES and FES. The system consists of lower leg and thigh cuffs with stimulators, a gait sensor, and a control unit that communicates by radio frequency signals. In the present study, the lower leg cuff was not used and the electrodes of the thigh cuff (two oval cloth electrodes, proximal: 130 mm *×* 75 mm; distal: 120 mm *×* 63 mm) were positioned over the quadriceps (Fig. 2). A biphasic symmetrical rectangular pulse waveform was applied, phase duration was 300 μsec, and stimulation frequency was 40 Hz. The stimulator provided a maximum intensity of 100 mA. The intensity of stimulation was increased by the physical therapist at each session and throughout all the sessions, in accordance with patient tolerance, to maximize quadriceps motor unit recruitment. Patients were encouraged to voluntarily activate their quadriceps muscle throughout the training.

Participants in the FES group received FES to the quadriceps for 10 min three days a week, while walking, in addition to the standard rehabilitation protocol. Computerized dynamic gait tracking algorithm analyzed the gait sensor’s data, and then transmitted the information to the stimulation unit to synchronize the quadriceps stimulation in accordance with the timing of gait events. The therapist used a hand-held computer to set the timing of the stimulation. To adjust the stimulation timing, stance and swing phases are presented to the clinician on the computer screen at 5% resolution. The quadriceps stimulation usually started with heel contact and terminated at the end of loading response (i.e., 20% of gait cycle) to provide greater confidence in shifting weight to the involved limb. In some patients, the clinician extended the stimulation toward mid-stance (i.e., 30% of gait cycle) to increase knee stability. The duration of this “extended” period is defined by percentage of the stance period.

Subjects in the NMES group used the same electrical stimulation system (i.e., identical electrode placement and stimulation parameters) while applying its NMES training mode. NMES training was performed for 10 min, 3 days a week, in addition to the standard rehabilitation protocol. The duty cycle was 10 s stimulation with 10 s pause.

### Assessments

To assess the feasibility of using FES, data on the ability to use FES in the first postoperative week according to the protocol, user acceptance, and adverse events were analyzed.

Outcome measures for efficacy were gait speed, gait symmetry, quadriceps isometric peak strength ratio, and peak strength symmetry. Outcomes were evaluated up to 2 weeks before the ACLR (pre-ACLR) and 4 weeks postoperatively (4-wks). An additional assessment of gait outcomes was performed 1 week after surgery (1-wk).

Gait speed was measured using the 10-m walk test (10MWT). During this test, the subjects were instructed to walk at a self-selected comfortable speed. The test was performed twice, and the average speed was used for data analysis.

Gait symmetry was evaluated while the subjects walked on a treadmill for 6 min at the average walking speed obtained from the 10MWT. The single limb stance percentage of gait cycle was measured using the OPTOGait system (Microgate, Bolzano, Italy). The OPTOGait system consists of a transmitter and receiver bars, each 1 m long, located on both sides of the treadmill. The transmitter bar has 99 infrared LEDs and the receiver bar has 99 sensors. Stepping between the bars blocks the infrared rays, allowing the system to obtain spatio-temporal gait parameters without the use of additional markers. Data were sampled at 1000 Hz and processed using dedicated software (Optojump Next, Version 1.3.20.0, Microgate, Bolzano, Italy). Gait symmetry was calculated using the equation: single limb stance of operated limb/single limb stance of non-operated limb.

The maximum voluntary isometric contraction torque was used to determine quadriceps strength. Measurements were performed using the Biodex Multi-Joint System (Biodex Corp, NY, USA). The patients were seated and stabilized with their knee flexed at 65°. They were instructed to maximally contract the quadriceps femoris muscles for 5 s while receiving verbal encouragement from the tester and visual feedback from the dynamometer. The patient had the opportunity to become accustomed to the test by performing up to 5 submaximal practice contractions. Maximum peak strength was defined as the highest peak (Newton-meters, Nm) obtained in a series of 5 attempts and was used for further data analyses. The ratio between the peak strength of the isometric quadriceps’ contraction of the operated limb at 4-wks to peak strength of the same limb pre-ACLR was quantified. Peak strength symmetry was calculated as the ratio between the operated and non-operated limb.

### Data analysis

Descriptive statistics was used to report the feasibility outcomes (e.g., ability to use FES in the first postoperative week), as well as participants’ baseline characteristics. The Shapiro-Wilk test was used to test the distribution of all continuous numeric variables. The t-test was used to compare baseline characteristics (age, height, and weight) between the FES and NMES groups, the pre-ACLR peak isometric quadriceps contraction strength of the operated limb, as well as to compare the quadriceps peak strength 4 weeks/pre-ACLR ratio. Repeated-measures, one-way ANOVAs were used to analyze the effect of treatment group and time on gait speed, gait symmetry, and peak strength symmetry (inter-limb). ANOVAs were followed by post hoc analyses with Bonferroni corrections, as appropriate. Cohen’s *d* was calculated to estimate the effect size of variables that were found to be significantly different between groups, 0.2 to 0.5 indicates a small effect, 0.5 to 0.8 indicates a moderate effect, 0.8 to 1.2 indicates a large effect, 1.2 to 2.0 indicates a very large effect, and > 2.0 indicates a huge effect [23]. Significance was determined as *P* < 0.05. The analysis was conducted using IBM SPSS, V23 (SPSS, Inc., Chicago, Illinois).

## Results

Baseline demographic data are presented in Table 1. There were no differences in age, height, or weight between groups. The sample included men only.

**Table 1:** Baseline demographic data

Variable	FES (*n* = 10)	NMES (*n* = 13)	*p*-value
Age (years)	20.4 ± 1.07	21.6 ± 4.17	0.381
	(19–22)	(19–30)	
Height (cm)	178 ± 8.5	175 ± 8.6	0.351
	(163–190)	(158–187)	
Weight (kg)	72.3 ± 6.7	70.6 ± 14.8	0.743
	(60–83)	(45–96)	
Gender male/female	10/0	13/0	___

Data are mean ± standard deviation and range in parentheses

### FES feasibility

All patients in the FES group were able to walk with the FES immediately after adjusting the stimulation and timing parameters. The patients were enthusiastic about using the system during rehabilitation, and there were no deviations from the course of rehabilitation or any adverse events related to the FES intervention.

### Efficacy outcomes

Table 2 summarizes the results of gait (speed and symmetry) and quadriceps strength (inter-limb symmetry, operated limb 4-wks/pre-ACLR ratio) outcomes, as well as the results of the analyses that tested the effects of group and time.

**Table 2:** Gait (speed and symmetry) and quadriceps strength (inter-limb symmetry, 4-wks/pre-ACLR ratio) outcomes, and effects of group and time

Variable	Time	FES (n = 10)	NMES (n = 13)	Time effect	Group effect	Time x Group effect
Gait speed (m/sec)	Pre-ACLR	1.16 ± 0.21	1.20 ± 0.22	< 0.001	0.344	0.023
		(95% CI 1.03–1.29)	(95% CI 1.08–1.32)			
	1-wk	0.89 ± 0.17	0.77 ± 0.27			
		(95% CI 0.78–1.0)	(95% CI 0.6–0.94)			
	4-wks	1.26 ± 0.13	1.11 ± 0.27			
		(95% CI 1.17–1.34)	(95% CI 0.96–1.26)			
Gait symmetry *operated/non- operated limb* (ratio)	Pre-ACLR	1.01 ± 0.03	1.00 ± 0.04	< 0.001	0.500	0.259
		(95% CI 0.99–1.03)	(95% CI 0.98–1.02)			
	1-wk	0.93 ± 0.08	0.93 ± 0.08			
		(95% CI 0.88–0.98)	(95% CI 0.87–0.99)			
	4-wks	1.01 ± 0.03	0.97 ± 0.06			
		(95% CI 0.99–1.03)	(95% CI 0.94–1.0)			
Peak strength *4-wks/pre-ACLR* (ratio)	4-wks	0.82 ± 0.27	0.47 ± 0.17	–	0.020	–
		(95% CI 0.64–0.99)	(95% CI 0.37–0.56)			
Peak strength symmetry *operated/non- operated limb* (ratio)	Pre-ACLR	0.86 ± 0.20	0.74 ± 0.25	< 0.001	0.038	0.040
		(95% CI 0.73–0.98)	(95% CI 0.6, 0.88)			
	4-wks	0.63 ± 0.15	0.39 ± 0.18			
		(95% CI 0.53–0.73)	(95% CI 0.29–0.49)			

#### Gait speed

Significant effects were found for time (*p* < 0.001) and time * group (*p* = 0.023). The post hoc analysis indicated that both groups regained their pre-ACLR speed after 4 weeks of rehabilitation. No other differences were found between groups.

#### Gait symmetry

A significant effect was found for time (*p* < 0.001) only.

#### Quadriceps 4 weeks/pre-ACLR peak strength ratio

Pre-ACLR peak strength was similar between groups (205 Nm FES vs. 225 Nm NMES, *p* = 0.605). Yet, quadriceps 4 weeks/pre-ACLR peak strength ratio was significantly better in the FES group as compared to the NMES group (0.82 ± 0.27 vs. 0.47 ± 0.17, p = 0.02), with a very large Cohen’s *d* effect size of 1.56. Meaning that after four weeks, subjects in the FES group regained 82% of their pre- quadriceps strength, while subjects in the NMES group regained only 47% of their pre-quadriceps strength.

#### Peak strength symmetry (operated/non-operated limb)

Significant effects were found for time (*p* < 0.001), group (0.038) and time * group (*p* = 0.040). The post hoc analysis indicated no difference between the FES and NMES groups pre-ACLR (0.86 ± 0.20 vs. 0.74 ± 0.25 *p* = 0.400). However, after 4 weeks of rehabilitation, the FES yielded significantly better inter-limb strength symmetry of 0.63 ± 0.15 compared to 0.39 ± 0.18 in the NMES group (*p* = 0.01), with a very large Cohen’s *d* effect size of 1.43. Indicating that after four weeks, compared to the non-operated limb, the strength deficit in the NMES group was of 61% and only 37% in the FES group.

**Fig. 3:**
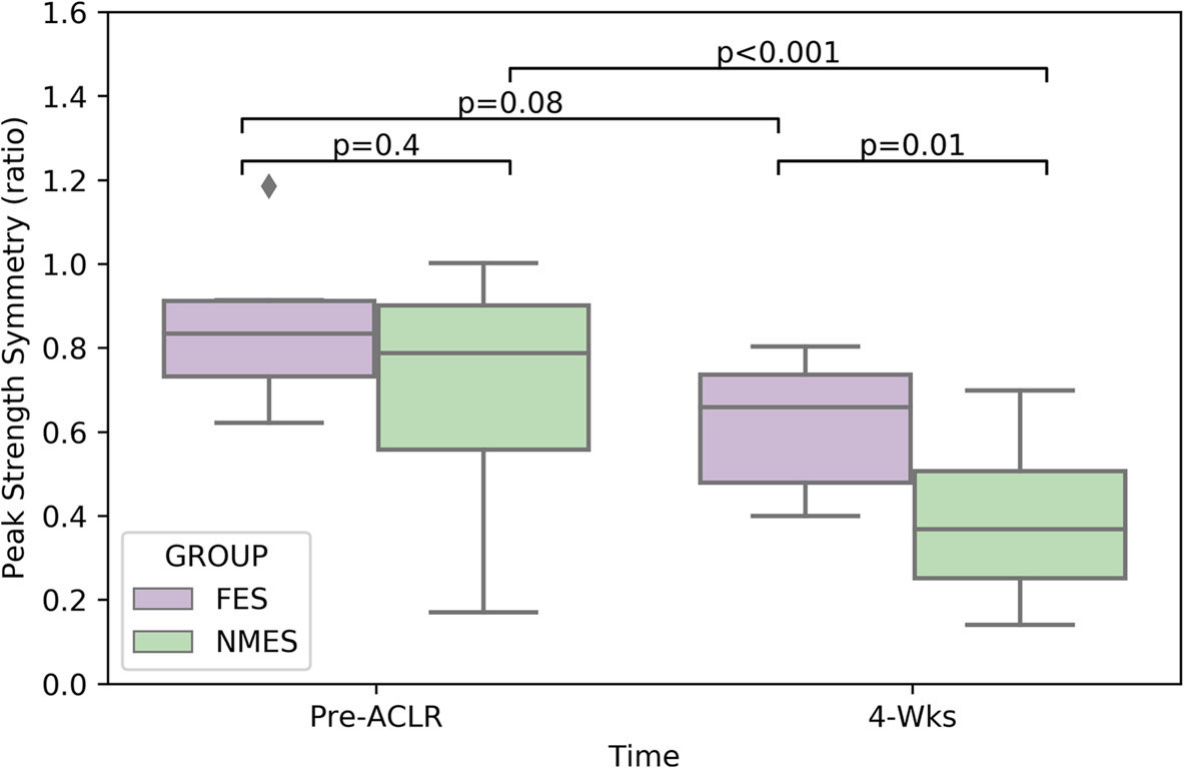
Peak strength symmetry (operated/non-operated limb)

Furthermore, as depicted in Fig. 3, the FES group regained pre-ACLR strength symmetry (*p* = 0.08), while the NMES group did not (*p* < 0.001).

## Discussion

The findings of this study demonstrated that quadriceps FES adjutant to standard rehabilitation is a feasible treatment option, early post-ACLR. Furthermore, at 4 weeks after surgery, quadriceps FES with traditional rehabilitation was more effective in recovering quadriceps muscle strength and symmetry than was NMES with traditional rehabilitation. These results are very important, as strong evidence now indicates that symmetrical quadriceps strength is an important rehabilitation target that significantly reduces the rate of post-ACLR knee reinjury [24].

The beneficial effects of FES on quadriceps strength may be explained by the implementation of motor learning principles that may help patients regain motor control and decrease AMI. Important features of motor learning are choosing task-specific training that is relevant and meaningful for the patients, incorporating repetitive practice, and enabling variability of practice [25]. These principles all were met by the quadriceps FES gait training. Gait retraining is essential component of ambulation post-ACLR and is associated with patient satisfaction [26]. Moreover, gait is probably the most important movement that patients should acquire during initial rehabilitation. Practicing gait involves many repetitions of quadriceps contractions in functional closed-kinetic chain and natural load. Gait training allows task variability, such as walking at different speeds and step lengths. Therefore, synchronous quadriceps motor unit recruitment achieved in functional movements of gait, probably contributed to the better quadriceps muscle function.

Our findings are consistent with those of previous studies, indicating that electrical stimulation coupled with movement may improve motor control even among able-bodied individuals [27–29]. For example, in a study protocol in which FES was used during walking, Thompson and Stein [27] measured cortical excitability (recorded by motor evoked potentials) of tibialis anterior and soleus muscles before and after 30 min of treadmill walking, with and without peroneal FES. They found motor evoked potentials increased in the tibialis anterior immediately after walking training with FES that lasted for 30 min. No significant increase was observed after walking without stimulation.

It should be also noted that while patients in both groups were instructed to voluntarily activate their quadriceps throughout the training, subjects in the NMES group probably focused on the muscle while contracting it, while subjects who used FES focused more on gait. Previous studies have suggested motor learning improves when there is an external focus of attention, also referred to as implicit learning [30, 31]. It may be assumed that external focus (i.e., on gait) helped to increase quadriceps motor control in the FES group.

While quadriceps strength and symmetry differed between groups, gait speed and symmetry did not. After 4 weeks of rehabilitation, both interventions were effective in improving gait outcomes compared to 1-week after surgery. These findings are consistent with a previous report [32] that demonstrated improvement in temporal gait variables accompanied by increased quadriceps strength following electrical stimulation. In this preliminary study, gait was assessed under comfortable, self-selected speed, and only spatial-temporal parameters were used to evaluate gait. Assessments with more challenging tasks, such as dual-task or fast walking, and evaluation of gait kinematics, kinetics, and electromyography may provide more comprehensive gait evaluation. Future research should assess the efficacy of quadriceps FES on varied gait outcomes in patients after ACLR. Furthermore, while strength is a direct measure of quadriceps function, gait function may be influenced by other residual impairments such as pain and stiffness that were not controlled for in the present protocol.

Finally, while both groups enhanced their walking abilities, it might have been that a different FES protocol would have resulted in increased control of gait. Most studies that applied NMES post-ACLR used a duty cycle of approximately 1:3 [12, 13]. In this study, in order to simulate the duty cycle of FES during gait, NMES was applied in a duty cycle of 1:1. Consequently, total treatment duration in both groups was limited to 10 min. It should be noted that 10 min of FES is much shorter compared to studies that applied FES for gait training [16]. Additionally, the frequency of electrical stimulation treatment sessions per week differed greatly in studies that used this method for muscle strengthening post-ACLR [13]. While Fitzgerald et al. [33] applied electrical stimulation for 2 treatment sessions per week, Delitto et al. [34] used it 5 days per week. Future investigations should also examine various protocols for FES application post-ACLR.

This study found that quadriceps FES may improve outcomes post-ACLR. It would also be interesting to investigate the effect of this intervention with subjects who underwent other surgical procedures to the knee, as well as to test the effect of FES training before surgery. Furthermore, to the best of the authors’ knowledge, this is the first report of FES during gait to rehabilitate musculoskeletal conditions. Based on the promising results of this report, it may be relevant to apply this method to other muscles for musculoskeletal conditions that are affected by AMI during walking, such as ankle musculature of patients with chronic ankle instability [35]. Yet, while FES systems are becoming more common in many rehabilitation centers, they are not available in most clinical settings. Therefore, further study into the cost-effectiveness of FES treatment for musculoskeletal conditions is warranted.

A limitation of this preliminary study was the relatively small sample size that included only men. Future studies should investigate the effects of quadriceps FES with larger samples that include female patients. Another limitation was the lack of an additional control group that would have received standard rehabilitation alone, without any form of electrical stimulation. However, implementation of this protocol might have been denied due to ethical issues, as electrical stimulation applied to the quadriceps is a recommended clinical practice following ACLR. It is also possible that factors related to motivation, due the interactive nature of FES use, have contributed to the improved results of the FES group. Finally, this study evaluated the effects of quadriceps FES during initial ACLR rehabilitation. Additional studies are needed to determine whether the effects of this intervention are sustained for a longer term.

## Conclusions

This study demonstrates the feasibility of applying quadriceps FES during walking as a new method to overcome quadriceps muscle weakness during early rehabilitation post-ACLR. Compared to NMES, patients who received FES achieved better results in quadriceps strength and symmetry. While both interventions were effective for the recovery of gait speed and symmetry, further investigations should evaluate the effects of quadriceps FES on kinetic and kinematic aspects of gait and other functional performance. In addition, the results of this preliminary study may encourage future research that will test the effectiveness of FES during gait for the rehabilitation of other relevant musculoskeletal conditions**.**

## Data Availability

The datasets used and/or analyzed during the current study are available from the corresponding author on reasonable request.

## Abbreviations

10MWT: 10-m walk test
ACLR: Anterior cruciate ligament reconstruction
AMI: Arthrogenic muscle inhibition
FES: Functional electrical stimulation
NMES: Neuro-muscular electrical stimulation
ROM: Range of motion
